# Isolated Basilic Vein Thrombosis as a Rare Presentation of COVID-19 in a Young Patient

**DOI:** 10.7759/cureus.14178

**Published:** 2021-03-29

**Authors:** Sameer Acharya, Janette Lee, Tyler Kelly, Binay K Kshetree

**Affiliations:** 1 Internal Medicine, Cayuga Medical Center, Ithaca, New York, USA

**Keywords:** hypercoagulopathy, covid-19 pandemic, deep vein thrombosis (dvt), thromboprophylaxis, thromboinflammation, upper arm dvt, anticoagulant prophylaxis

## Abstract

COVID-19 has not spared a single system in the human body. Although acute respiratory failure culminating sometimes in death remains the most common manifestation of severe infection, hypercoagulability leading to deep vein thrombosis (DVT), pulmonary embolism (PE), and stroke have also been identified widely. Here, we describe a young patient with a COVID-19 infection who developed right basilic vein thrombosis. This case demonstrates how thrombosis can occur in uncommon sites and how clinicians should be vigilant for thrombotic complications in both the inpatient and outpatient settings.

## Introduction

COVID-19, at the onset of the global pandemic, was initially recognized as primarily a pulmonary infection, but quickly encompassed a spectrum of system diseases that have yet to be fully understood. Although acute hypoxic respiratory failure [1] leading to advanced lung disease and death remains the most recognized manifestation of severe infection, hypercoagulability has also been identified as an abnormal state caused by COVID-19 [2]. The pathogenesis of thrombosis is thought to be due to direct invasion of COVID-19 virus of endothelial cells via angiotensin-converting enzyme 2 expressed on the cell surface [3]. The subsequent endothelial inflammation, complement activation, thrombin generation, platelet, and leukocyte recruitment, and the initiation of innate and adaptive immune responses result in thromboinflammation, ultimately leading to complications such as deep vein thrombosis (DVT), pulmonary embolism (PE), and stroke [4]. While the most common manifestations of hyper-coagulopathy in the setting of severe COVID-19 infection have been DVT and PE, upper extremity thrombosis has been only rarely reported [5]. Here, we present an isolated right basilic vein thrombosis in a morbidly obese young man with a COVID-19 infection.

## Case presentation

A 22-year-old-male with a past medical history of morbid obesity, who was exposed to COVID-19 from close contact, reported nausea, dyspnea on exertion, and diarrhea which began approximately six days after exposure; he presented to the emergency department with two days of headache, fever (38.9 °C), and right upper arm pain and swelling.

His vital signs on presentation were: blood pressure 132/83 mmHg, pulse 95 bpm, respirations 24 breaths per minute, oxygen saturation 80% on room air, temperature 35.6 °C. His body mass index was 46.1 kg/m^2^. The right upper arm was mildly tender, with minimal swelling in the medial aspect, without skin changes. The remainder of his examination was largely normal. Baseline 12-lead electrocardiogram was normal. Arterial blood gas showed hypoxemia, with paO_2_ 69 and oxygen saturation in the 80s, necessitating that he be placed on supplemental oxygen at 2 L. 

**Figure 1:**
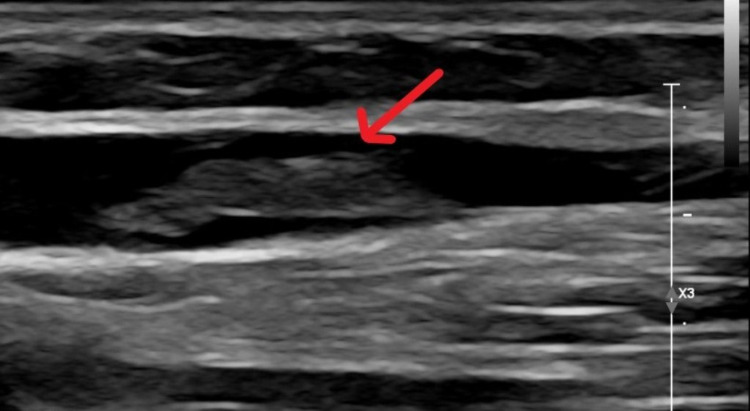
Ultrasound of right upper arm showing thrombus in basilic vein.

His complete blood count, basic metabolic panel, alkaline phosphatase, and d-dimers were normal; he did have mildly elevated transaminases, C-reactive protein of 13.76 mg/L, and an international normalized ratio of 1.75. He was treated with supplemental oxygen, intravenous Dexamethasone, Remdesivir, and subcutaneous Enoxaparin for five days. Ultrasound of his right upper extremity revealed a non-occlusive thrombus in the basilic vein (Figure 1) and a non-visualized cephalic vein with possible chronic thrombosis.

He was placed on oral Apixaban 5 mg twice daily for a planned duration of three months, as per guidelines [6]. His right upper arm pain and swelling subsequently resolved on a follow-up visit after 14 days and his repeat d-dimer remained normal.

## Discussion

Lower-extremity DVT has been a commonly reported complication of COVID-19, recently [1] ascribed to both prolonged hospital stay as well as COVID-19-induced hyper-coagulopathy [5]. We have also seen multiple reports of extensive microthrombi culminating in PE, stroke, or myocardial infarction. Although arterial thrombosis causing acute upper limb ischemia in the setting of COVID-19 infection has been reported [7,8], isolated upper-extremity DVT is a rare case presentation [5]. Our report aims to highlight the possibility of an insidious onset of upper extremity DVT in the context of COVID-19 infection with less severe manifestations, in a young patient. A broader discussion is warranted about how this should impact our practice. While there are guidelines adopted widely and locally regarding appropriate thromboprophylaxis in hospitalized patients with COVID-19, there are no recommendations to use either antiplatelet agents or anticoagulation in the outpatient with COVID-19 (nor are their data to support such a practice.) However, this unusual case suggests that clinicians should at least be more vigilant with COVID-19 infected patients who are at higher risk for thrombosis.

## Conclusions

COVID-19 has challenged clinicians with its protean clinical manifestations and range of outcomes, from asymptomatic to multiple-system decompensation to death. Likewise, COVID-19-induced hypercoagulopathies present in widely differing manners, not just as life-threatening complications in the critically ill hospitalized patient, but also as an easily-overlooked thrombosis in an unexpected region of the body in the out-patient setting. Although there are no current trials or guidelines that suggest that routine thromboprophylaxis is appropriate in outpatients with COVID-19, it appears that close monitoring, especially of individuals at increased risk for thrombosis, would be a prudent approach. Moreover, as clinical signs of thrombosis may be subtle, as in our case, clinicians should have a low threshold for further investigation in the setting of COVID-19.
